# Expression of transforming growth factor β and its correlation with lipodystrophy in oral submucous fibrosis: An immunohistochemical study

**DOI:** 10.4317/medoral.18226

**Published:** 2012-08-28

**Authors:** Alka D. Kale, Deepa R. Mane, Deepika Shukla

**Affiliations:** 1 BDS, MDS. Department of Oral Pathology and Microbiology, KLE VK Institute of Dental Sciences and Hospital, Belgaum, Karnataka, India; 2 BDS, MDS. Department of Oral Pathology and Microbiology, KLE VK Institute of Dental Sciences and Hospital, Belgaum, Karnataka, India; 3 BDS, MDS. Department of Oral Pathology and Microbiology, Faculty of Dentistry, Jamia Millia Islamia, Delhi, India

## Abstract

Objective: In our routine review of Oral Submucous Fibrosis (OSMF) biopsies, we observed decreased adipose tissue even though most are from buccal mucosa. Pathogenesis of OSMF has demonstrated the role of Transforming Growth Factor β (TGF β), in causing fibrosis. This study aims to correlate the role of TGF β with loss of adipose tissue in OSMF. Study Design: From our archives, 84 OSMF cases (24 early and 60 advanced OSMF) were screened for adipose tissue. Immunoexpression of TGF β in these cases were investigated. Results: Adipose tissue was seen in 67% of early OSMF and in 13% of advanced cases. Early cases showed more intense TGF β staining of epithelium, fibroblast, macrophages and inflammatory cells than the advanced cases. Conclusion: These findings suggest that TGF β plays a key role in causing lipodystrophy in OSMF and is secreted more during early course of the disease than in advanced stage.

** Key words:**Adipose tissue, oral submucous fibrosis (OSMF), transforming growth factor β (TGF β), lipodystrophy.

## Introduction

Oral Submucous Fibrosis (OSMF) is a high risk precancerous condition, predominantly affecting south East Asians. Studies have shown that none of the treatment approaches is completely effective in these patients and relapse is a common complication (1,2). Histologically, Pindborg and Sirsat described four consecutive stages depending upon hyalinization, fibroblastic response and inflammation (3). Further, Binnie and Cawson revealed degeneration of muscle fibers together with collagenous subepithelial zone (4). Pathogenesis of OSMF has explained the role of growth factors and cytokines that are secreted by inflammatory cells during the disease process which promotes fibrosis by inducing proliferation of fibroblasts, upregulating collagen synthesis and down regulating collagenase production (5). One such key molecule is Transforming Growth Factor β (TGF β) that is a central matrix modulator.

TGF β has been found to play role in regulation of cell growth, differentiation, proliferation, migration, adhesion and apoptosis (6). It causes increased proliferation of fibroblasts (7) but inhibits proliferation of epithelial cells; causes differentiation of neuronal cells, but blocks differentiation of mesenchymal cells (8). TGF β isoforms exhibit overlapping but distinct temporal and spatial patterns of expression in vivo. TGF β1 is expressed in epithelial, hematopoietic, and connective tissue cells, TGF β2 in epithelial and neuronal cells and TGF β3 primarily in mesenchymal cells (9).

In OSMF, TGF β is a key mediator of tissue fibrosis resulting from accumulation of extra cellular matrix (ECM). Its activator protein induces transcription of COL1A1 procollagen gene (9–11), increases levels and activities of the N–and C–procollagen proteinases (12) and promotes the expression of lysyl oxidase (LOX), an essential enzyme for final processing of collagen fibers into a stabilized covalently cross–linked mature fibrillar form that is resistant to proteolysis (13,14). TGF β also decreases the collagen degradation by activating tissue inhibitor of matrix metalloproteinase gene (TIMPs) and plasminogen activator inhibitor (PAI) gene (12). Although transient TGF β1 activity participates in repair and regeneration of tissues, persistent TGF β 1 function effects excessive fibrosis (11). TGF β causes induction of connective tissue growth factor (CTGF), which further mediates stimulatory actions of TGF β on ECM synthesis (15). It also initiates fibrosis in skeletal muscle and induces myogenic cells to differentiate into myofibroblastic cells in injured muscle (16).

TGF β1 has been implicated in lipodystrophy as demonstrated by Clouthier DE et al (17). Nevertheless there is a paucity of information related to adipose tissue in OSMF. Over a period of years of our histopathological observation of OSMF cases, significant absence of adipose tissue was noted. Could the destruction of adipose tissue by TGF β be responsible for the clinical, facial and oral appearance of OSMF? This study aims to establish if there is any association of degeneration of adipose tissue and TGF β. This information would potentially be useful in identifying those OSMF cases in which replacement of adipose tissue or supplementing anti TGF β drugs could lead to better prognosis.

## Material and Methods

-Case Selection

Eighty four formal infixed paraffin embedded tissue blocks of histopathologically cases of OSMF were retrieved from the archives of the Department of Oral and Maxillofacial Pathology, KLE VK Institute of Dental Sciences, Belgaum for this study. Institutional Review Board and Ethical Committee approval was obtained prior to the start of the study. The KLE VK Institute of Dental Sciences actively maintains case histories of all the patients for clinical findings. Case histories were evaluated for clinical findings like burning sensation, ulceration, trismus, pale buccal mucosa firmly attached to underlying tissues, bands of palpable fibrosis and sunken cheek appearance. Haematoxylin and Eosin (H & E) sections of these OSMF cases were reviewed by three oral pathologists and categorized into very early, early, moderately advanced and advanced OSMF based on classification of Pindborg and Sirsat (3). The very early stage OSMF is characterized by finely fibrillar collagen, plump young fibroblasts, inflammatory cells and dilated blood vessels. The early stage shows early juxtaepithelial hyalinization with separate collagen bundles. In moderately advanced stage collagen is moderately hyalinized with thickened collagen bundles still separated by slight residual edema. In the advanced stage, collagen undergoes complete hyalinization with no distinct bundles, absence of fibroblasts and obliterated blood vessels. For ease of evaluation we have classified the cases into two groups (a) ‘early’ (24 cases) including very early OSMF and early OSMF and (b) ‘advanced’ (60 cases) including moderately advanced OSMF and advanced OSMF. Immunohistochemical staining with TGF β was done for these OSMF cases. Desmoplastic ameloblastoma, fibroma and chronic inflammatory hyperplasia were included in the study to assess TGF β expression in inflammatory and fibrosed tissues.

-Immunohistochemistry Protocol

Formalin fixed, paraffin embedded serial tissue sections cut at 5 µm were deparaffinized and subjected to immunohistochemical technique using Super SensitiveTM polymer-HRP Detection System (BIOGENEX Corporations, San Ramon, CA 94583, USA). Heat induced epitope retrieval was done in EZ Antigen retrieval system (Biogenex) following 3 cycles of 96ºC for 6 min each placing slides in sodium citrate buffer (pH 6.0) and finally allowing it to cool at room temperature for 20 min. Endogenous peroxidase was blocked with 3% hydrogen peroxide in water for 15 min. The sections were then incubated with protein blocking reagent for 20 min at room temperature to block the non–specific binding sites. The sections were then incubated with a primary antibody against TGF β (Anti TGF β1, β2, β3, 1D11 from R&D Systems, 1:200 dilutions) at 4ºC for overnight in a humidifying chamber. Following this, all sections were washed with PBS again (three times, 10 min each) and then incubated with secondary antibodies that were conjugated with poly-horseradish peroxidase reagent for a further 20 min. Bound peroxidase was visualized by a 3,3’diaminobenzidine hydrochloride and counter stain was developed with Harris hematoxylin. Phosphate buffered saline of pH 7.6 was used throughout for washing and rinsing the slides. To determine the specificity of the antibodies, phosphate–buffered saline was used instead of primary antibody and secondary antibody was omitted on some slides. The intensity of staining of the epithelium (basal and superficial) and stroma was assessed as: – negative (no staining); + mild (positive staining for less than one-third of tissue section) ; ++ moderate (positive staining area ranged from one-third to two-third of tissue section) and +++ intense (positive staining for more than two-third of tissue section). Three independent oral pathologists evaluated the slides and all observers were blinded.

-Data Analysis

Data were entered and analyzed using SPSS 10.0.5 software. The Chi–square test and Fisher’s test was used to analyze the differences in between the intensity levels and percentage positivity in early OSMF and advanced OSMF for TGF β positivity and to compare both the groups with respect to the presence of adipose tissue. Differences with a probability value of <0.05 were considered statistically significant.

## Results

-Clinical Presentation

In the present study, early OSMF cases (24 cases) were included which clinically presented with burning sensation, ulceration, widespread bands of palpable fibrosis and histopathologically showed finely fibrillar collagen or in separate thick bundles with fibroblasts, inflammatory cells and dilated and congested blood vessels. Advanced cases (60 cases) in this study presented with trismus, pale buccal mucosa firmly attached to underlying tissues giving a tough leathery feeling, vertical fibrous bands palpable at soft palate, pterygomandibular raphe, faucial pillars and sunken cheek appearance. All the patients had habit of areca nut chewing for more than 5 years.

-Haematoxylin and Eosin staining

**Figure 1:**
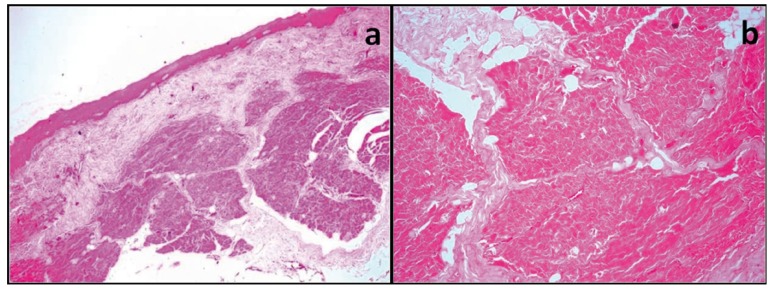
H&E Photomicrograph of a) early OSMF (original magnification 10x) and b) advanced OSMF (original magnification 40x) with absence of adipose tissue.

In H&E sections, all cases demonstrated thickened or completely hyalinized collagen bundles with atrophic epithelium, decreased cellularity (fibroblasts), compressed blood vessels and degeneration of muscle fibres. Amongst the early and advanced OSMF only 24 cases out of 84 showed presence of adipose tissue. In early OSMF cases, 33% (8 out of 24 cases) showed no adipose tissue while advanced cases 86.7% (52 out of 60 cases) showed lack of adipose tissue. These findings were found to be statistically significant with Chi-square analysis with p value being <0.01 ( Table 1, Fig. 1).

**Table 1:**
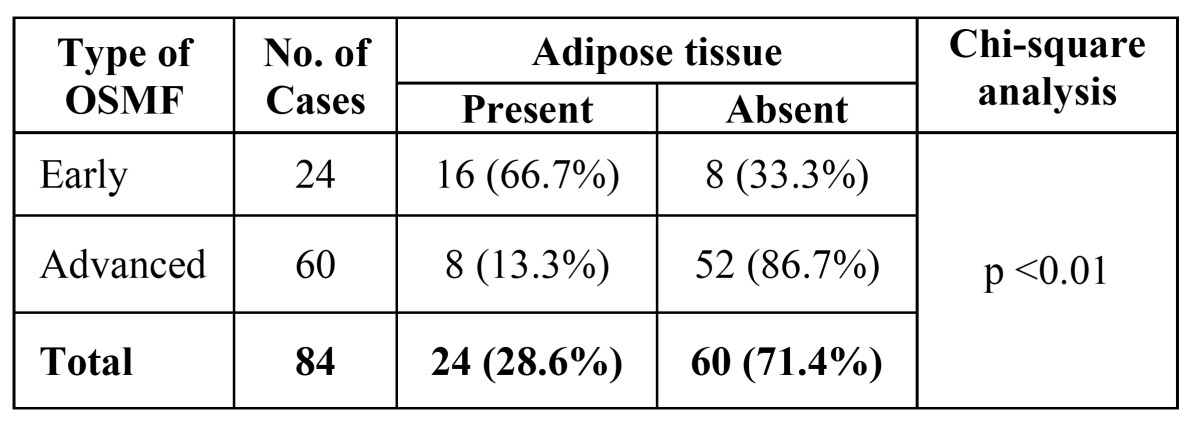
Distribution of adipose tissue in Early and Advanced OSMF (H&E). Data were entered and analyzed using SPSS 10.0.5 software. The Chi–square test and Fisher’s test was used to compare both the groups with respect to the presence of adipose tissue. These findings were found to be statistically significant with p value being <0.01.

-Immunohistochemical staining for TGF β

**Figure 2:**
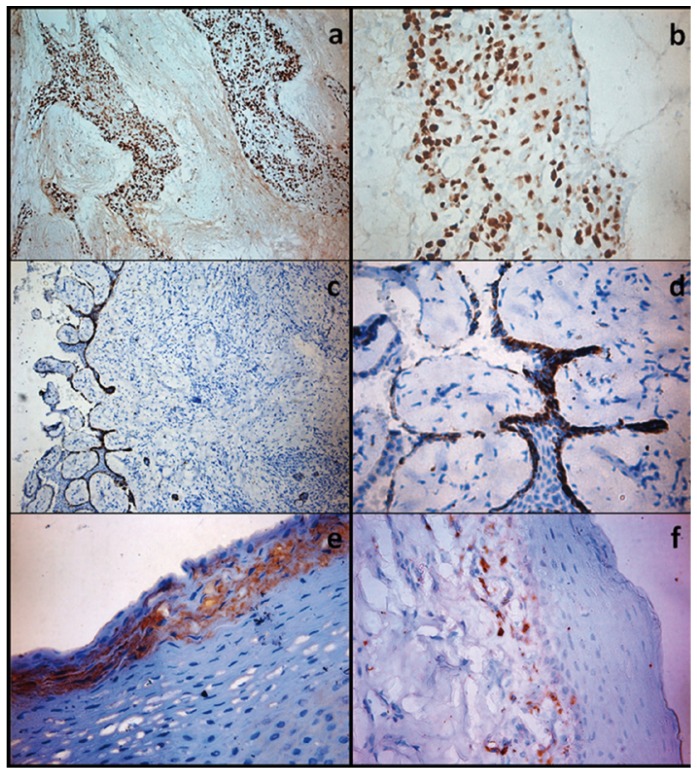
Photomicrographs showing positive intense type of immunohistochemical staining for TGF β (+ + +) in desmoplastic ameloblastoma a,b), moderate type of staining (+ + ) in chronic inflammatory hyperplasia c,d) and moderate to mild type of staining (+) in fibroma e,f) which are taken as positive control (a,c,e- original magnification 10x; b,d,f- original magnification 40x).

Desmoplastic ameloblastoma, fibroma and chronic inflammatory hyperplasia that served as positive controls showed strong TGF β positivity in all the cases (Fig. 2). These controls verified that the antigenic expression was properly preserved in the study group.

**Figure 3:**
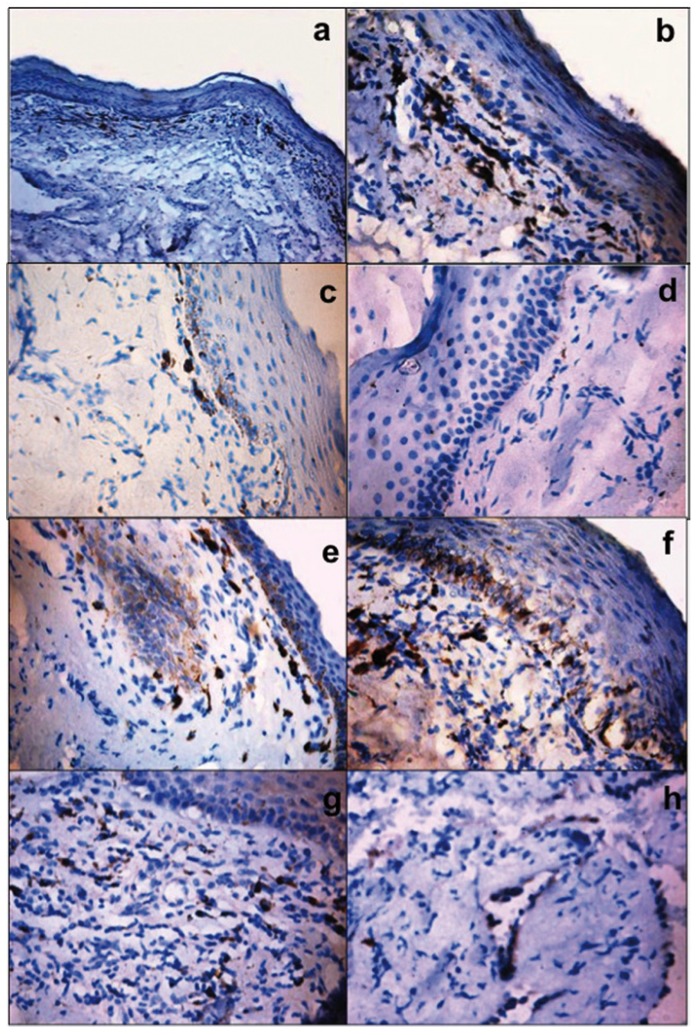
Immunohistochemistry for TGF β expression in early OSMF a,b) and advanced OSMF c,d). Photomicrographs are showing intense type of staining for TGF β in early OSMF while only mild TGF β expression in advanced OSMF cases. Immunohistochemistry for TGF β expression in epithelium and stroma e,f) and stromal cells g,h) of OSMF. Photomicrographs are showing intense staining of basal and superficial epithelium in early OSMF where as mild to moderate staining in advanced OSMF cases. Fibroblast, inflammatory cells and endothelial cells are also showing positive staining (a,e,g- original magnification 10x; b,c,d,f,h- original magnification 40x).

In TGF β stained sections, out of 24 cases of early OSMF (Fig. 3), 22 cases (92%) showed mild to intense type of staining intensity for TGF β while only two cases showed no staining. Out of 60 advanced cases of OSMF, 34 cases (57%) showed mild to moderate TGF β expression, where as 26 cases (43%) had no staining for TGF β ( Table 2, Fig. 3).

**Table 2:**
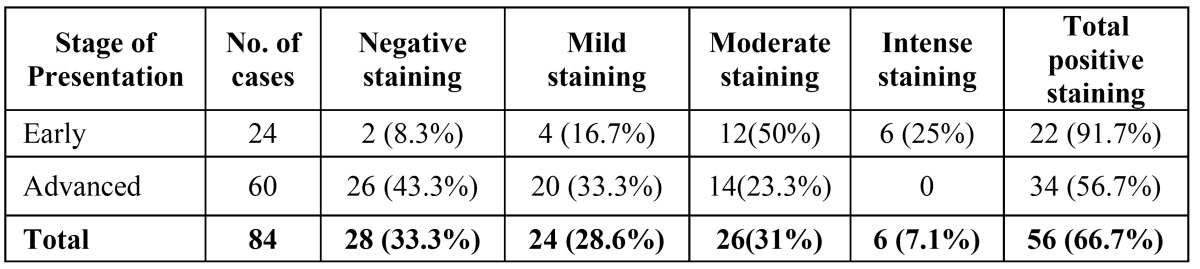
Intensity of TGF β expression in OSMF (IHC).

Expression of TGF β was evaluated in the epithelium, stromal cells and in the stromal tissue of OSMF. Out of the 56 OSMF cases positive for TGF β expression, 18 cases (32%) showed TGF β expression in superficial layers of epithelium where as 31 cases (55%) showed expression in basal layers and 7 cases (13%) showed expression in both superficial and basal layers ( Table 3). Of the 56 cases of TGF β positive expression, 49 cases showed positivity for fibroblast, inflammatory cells and endothelial cells and 28 cases demonstrated mild to moderate expression in deeper stroma ( Table 4, Fig. 3). There was no statistical significant difference in relation to TGF β expression in early and advanced OSMF or in its expression in superficial and basal layer of epithelium, stromal cells and also in deeper stroma.

**Table 3:**
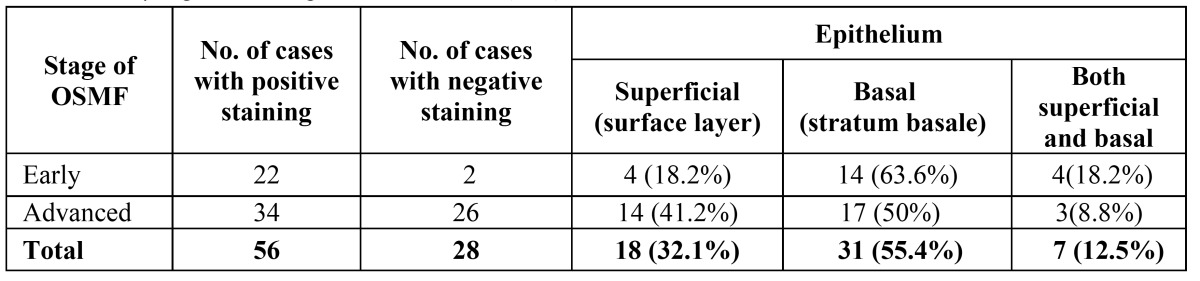
TGF β expression in epithelium of OSMF (IHC).

**Table 4:**
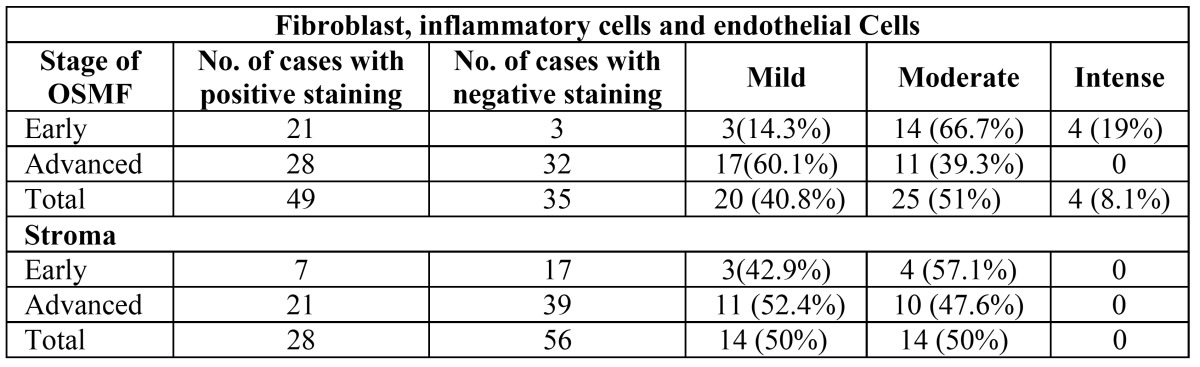
Intensity of TGF β expression in fibroblast, inflammatory cells, endothelial cells and stroma in OSMF (IHC).

## Discussion

OSMF is an oral disease first described three decades ago by Pindborg and Sirsat who graded it histologically into four stages depending upon hyalinization, fibroblastic response and inflammation (3). OSMF has been regarded as a collagen–metabolic disorder. Increased collagen production and decreased collagen degradation results in increased collagen deposition in the oral tissue, leading to fibrosis. This is further aggravated by the auto-regulatory process of TGF β, which is the main trigger for both the increased collagen production and decreased degradation pathways and recently has also been found to be responsible in inhibiting adipogenesis. Understanding of the molecular events helps in better therapeutic intervention of the disease. Thorough literature search through Pubmed and Medline could not reveal any data regarding absence of adipose tissue in OSMF. Thus, this study aimed to correlate the absence of fatty tissue observed in OSMF patients with the underlying molecular events by targeting TGF β.

In the present study epithelium showed more positivity in basal layer than superficial layer. Further the intensity and frequency of staining was more in early OSMF than advanced OSMF cases ( Table 2, Table 3). Previous studies have found that external stimuli such as areca nut components may induce the development of OSMF by activating and stimulating the keratinocytes to secrete a series of cytokines, including endothelin (ET) and TGF β 1 (18). Interestingly, Gao Y et al have shown that there was positive expression of TGF β 1 mRNA mainly in keratinocytes of early and middle stage OSMF which is in agreement with the present study (19). This suggests that keratinocytes of OSMF tissue may synthesize and release TGF β 1 which may play an important role in the pathogenesis of OSMF and participate as a mediator in the pathogenetic process of OSMF (19,20).

Furthermore, stromal cells that are well known secretors of TGF β demonstrated better expression in early OSMF (88%) as compared to advanced OSMF (47%) though stromal staining was equally distributed in both groups ( Table 4). TGF β positivity was more in early OSMF (92%) cases than in advanced OSMF cases (57%) and adipose tissue also was seen more in early OSMF cases (67%) as compared to advanced cases (13%). Early cases showed increased intensity of staining of epithelium, fibroblast, macrophages and inflammatory cells than advanced cases. It was found that in response to external stimuli and inflammation, TGF β is secreted more during early course of the disease when widespread sheets of fibrous bands are palpated. This leads to increased fibrous proliferation and destruction of adipose tissue. TGF β decreases gradually in advanced cases when the patient presents with vertical fibrous bands and sunken cheek appearance, as the tissue has already fibrosed and lost all its adipose tissue

Previous studies have established that TGF β1 is a regulator of adipocyte differentiation and its expression in adipose tissue resulted in a lipodystrophy-like syndrome (17,21). TGF β1 may inhibit adipogenesis directly by modulating the expression of critically required molecules, or indirectly by enhancing ECM synthesis and deposition. It induces pathologic matrix accumulation in the adipose tissue, resulting in severe fibrotic disease. It is presumed that the severe fibrosis of the adipose tissue is the underlying cause of the loss of skin elasticity, thus creating a tight–skin phenotype (sunken cheek appearance) (17). In vitro studies have revealed that TGF β1 binds to receptors on the plasma membrane of fibroblasts (3T3–L1) and effectively inhibits their adipogenic conversion (21). It has been established that endogenous TGF β signaling regulates the rate of adipogenesis, and that Smad2 and Smad3 plays key role in this endogenous control of differentiation (22). TGF β activated Smad proteins interacts with enhancer binding proteins (C/EBP β or C/EBP δ) and thus inhibits adipocyte differentiation (23). Further Petruschke et al, found that addition of TGF β at the beginning of the differentiation process resulted in a dose–dependent reduced expression of glyceraldehyde–3–phosphate dehydrogenase (GPDH) activity. Consequently, cells were not able to accumulate lipid droplets, suggesting that TGF β inhibits human adipose tissue development and reduces the activity of a lipogenic key enzyme in newly formed fat cells (24).

Evaluating this literature, we have hypothesised that adipose tissue in OSMF could be reduced by over expression of TGF β. We demonstrated that TGF β expression in OSMF remains elevated as the disease progresses from Early to Advanced and there is a concomitant lack of adipose tissue in these cases. However, fibrosis in OSMF may still be derived from stromal cells and the decrease of fat cell could still be a consequence of inflammation. To definitively clarify a correlation between TGF β and fat content in OSMF, further studies with more samples are required. Adipose tissue is derived from the embryonic mesenchyme and contains a stroma that is easily isolated. Few studies have recently recognized a stem cell population within the adipose stromal compartment, termed processed lipoaspirate cells, which can differentiate toward the adipogenic, chondrogenic myogenic and osteogenic lineages (25-27). Preadipocytes are a feasible cell source for adipose tissue regeneration (28). Hence in future adipose tissue replacement could be used to treat OSMF cases. However, the enhancement of the in vivo adipogenic conversion of preadipocytes remains a major challenge.

This study thus suggests TGF β to be the molecule participating in lipodystrophy and inhibiting adipogenesis in OSMF. Loss of adipose tissue along with the fibrosis can be a cause of stiffness of oral mucosa and sunken appearance of cheeks in most OSMF cases. Further studies are required to investigate if replacement of adipose tissue and augmentation of lost soft tissues in such patients could aid as a treatment modality in advanced OSMF.
